# Prevalence and patterns of symptoms of dysautonomia in patients with long‐COVID syndrome: A cross‐sectional study

**DOI:** 10.1002/acn3.51557

**Published:** 2022-04-08

**Authors:** Ahmed M. Eldokla, Aliae A. Mohamed‐Hussein, Ahmed M. Fouad, Mariam G. Abdelnaser, Sara T. Ali, Nahed A. Makhlouf, Islam G. Sayed, Hoda A. Makhlouf, Jaffer Shah, Hani Aiash

**Affiliations:** ^1^ Department of Neurology, State University of New York Upstate Medical University Syracuse New York 13210 USA; ^2^ Department of Pathology, State University of New York Upstate Medical University Syracuse New York 13210 USA; ^3^ Chest Department, Faculty of Medicine Assiut University Assiut Egypt; ^4^ Department of Public Health, Occupational and Environmental Medicine, Faculty of Medicine Suez Canal University Ismailia Egypt; ^5^ Department of Public Health and Community Medicine Faculty of Medicine Assiut University Assiut Egypt; ^6^ Department of Tropical Medicine and Gastroenterology, Faculty of Medicine Assiut University Assiut Egypt; ^7^ New York State Department of Health Albany New York USA; ^8^ Department of Family Medicine Suez Canal University Ismailia Egypt; ^9^ Department of Cardiovascular Perfusion, State University of New York Upstate Medical University Syracuse New York 13210 USA

## Abstract

**Background:**

The association between autonomic dysfunction and long‐COVID syndrome is established. However, the prevalence and patterns of symptoms of dysautonomia in long‐COVID syndrome in a large population are lacking.

**Objective:**

To evaluate the prevalence and patterns of symptoms of dysautonomia in patients with long‐COVID syndrome.

**Methods:**

We administered the Composite Autonomic Symptom Score 31 (COMPASS‐31) questionnaire to a sample of post‐COVID‐19 patients who were referred to post‐COVID clinic in Assiut University Hospitals, Egypt for symptoms concerning for long‐COVID syndrome. Participants were asked to complete the COMPASS‐31 questionnaire referring to the period of more than 4 weeks after acute COVID‐19.

**Results:**

We included 320 patients (35.92 ± 11.92 years, 73% females). The median COMPASS‐31 score was 26.29 (0–76.73). The most affected domains of dysautonomia were gastrointestinal, secretomotor, and orthostatic intolerance with 91.6%, 76.4%, and 73.6%, respectively. There was a positive correlation between COMPASS‐31 score and long‐COVID duration (*p* < 0.001) and a positive correlation between orthostatic intolerance domain score and post‐COVID duration (*p* < 0.001). There was a positive correlation between orthostatic intolerance domain score and age of participants (*p* = 0.004). Two hundred forty‐seven patients (76.7%) had a high score of COMPASS‐31 >16.4. Patients with COMPASS‐31 >16.4 had a longer duration of long‐COVID syndrome than those with score <16.4 (46.2 vs. 26.8 weeks, *p* < 0.001).

**Conclusions:**

Symptoms of dysautonomia are common in long‐COVID syndrome. The most common COMPASS‐31 affected domains of dysautonomia are gastrointestinal, secretomotor, and orthostatic intolerance. There is a positive correlation between orthostatic intolerance domain score and patients' age.

## Introduction

COVID‐19 emerged in the late 2019 and since then it has been a wide‐spread epidemic with significant morbidity and mortality.^1^ After the world entered the battle of confronting acute COVID‐19, it was discovered that it does not end with early identification and management of the acute COVID‐19, and late health consequences have appeared among COVID‐19 survivors.^2^ It becomes obvious that the morbidity of COVID‐19 is not only limited to the acute infection, but it also extends behind the acute phase and causes long‐term sequelae described as “post‐acute COVID syndrome” (PACS) or “long‐COVID syndrome.” The United Kingdom National Institute for Health and Care Excellence (NICE) has defined the long‐COVID syndrome as signs and symptoms that develop from 4 to 12 weeks after the onset of illness and post‐COVID syndrome (12 weeks or more), and are not explained by an alternative diagnosis.^3^ Long‐COVID syndrome has been reported after mild or severe COVID‐19 irrespective of the severity of the acute phase of COVID‐19.^4^ Direct invasion of the virus into the brain by binding to angiotensin‐converting enzyme 2 (ACE2) expressed in the endothelium of the capillary of the blood–brain barrier or an indirect effect of the cytokine storm on mitochondria or on nerve fibers,^5^ immune dysregulation, hormonal disturbances, elevated cytokine levels due to immune reaction leading to chronic inflammation have been proposed, among others as possible pathophysiological mechanisms for developing neurological manifestations of long‐COVID.^6^

Long‐COVID syndrome encompasses many symptoms including shortness of breath, headaches, memory changes, nausea, anorexia, abnormal sweating, palpitation, anxiety, depression, fatigue, chest pain, and orthostatic intolerance (OI).^7^, ^8^, ^9^, ^10^, ^11^, ^12^ Many of these symptoms are seen in patients with autonomic dysfunction. Additionally, postural orthostatic tachycardia syndrome, OI, abnormal sudomotor function, and neuropathy have been reported to be associated or worsened with COVID‐19.^13^, ^14^, ^15^ Although the association between autonomic dysfunction and long‐COVID syndrome has gained considerable interest,^15^, ^16^ the prevalence of autonomic dysfunction has not well‐studied in long‐COVID population. Therefore, this study aimed to estimate the prevalence and describe the patterns of autonomic symptoms in long‐COVID patients.

## Methods

### Participants and settings

This cross‐sectional study was conducted on patients referred to the post‐COVID clinic at Assiut University Hospitals, Egypt. Patients were included in this study if they were above 18‐year‐old, symptomatic at SARS‐CoV‐2 infection acute phase regardless of the severity of the symptoms or the need for oxygen or ICU support, had a laboratory‐confirmed (polymerase chain reaction and/or antibody testing) diagnosis, and had symptoms of long‐COVID syndrome^8^ that developed or persisted at least 4 weeks after the onset of illness. Patients were excluded if they had a diagnosis of cognitive dysfunction, neurodegenerative brain disorders, known history of autonomic dysfunction, or were taking medications that significantly affect the autonomic nervous system (ANS) as beta‐blocker, tricyclic antidepressant, alpha‐1 blockers, and central antihypertensives.

A total of 356 patients had been referred to the post‐COVID clinic during the study period between 01 August 2021 and 01 November 2021. This number was large enough to detect an anticipated frequency of symptoms of dysautonomia among long‐COVID patients of 61.1% as reported in an earlier study,^17^ with a 95% confidence level and 5% absolute precision, given that the total number of the study population was <100,000. This calculation was performed using Epi Info™ Statistical Package version 7.2.4.0 (Centers for Disease Control and Prevention, Atlanta, GA, USA).

Eligible patients were retrospectively identified from the medical records of the post‐COVID clinic then invited for a clinical visit between 01 October 2021 and 01 November 2021 to complete data collection. Baseline demographic and clinical data (including acute COVID‐19 related data) were retrieved from patients' medical records. Missing information due to incomplete records was collected during their most recent visit to the post‐COVID clinic.

### COMPASS‐31 questionnaire

Furthermore, participants were asked to complete the Composite Autonomic Symptom Score 31 (COMPASS‐31) questionnaire, referring to the period after the COVID‐19 (at least 4 weeks following the onset of acute COVID‐19). The COMPASS‐31 is validated and widely used questionnaire to quantify autonomic symptom severity. It consists of 31 questions that fall into six domains of dysautonomia: OI, vasomotor, secretomotor, gastrointestinal (GI), bladder, and pupillomotor. An answer was scored as zero when it was not assigned a point. A raw domain score was obtained by adding together points within each domain. The total score within each domain was weighted and then added together to give a total score ranging from 0 to 100. The maximum‐weighted scores for each subdomain are as follows: 40 for OI, 5 for vasomotor dysfunction, 15 for secretomotor dysfunction, 25 for GI dysfunction, 10 for urinary dysfunction, and 5 for pupillomotor dysfunction.^18^ A total COMPASS‐31 of >16.4 was used to suggest initial autonomic nervous system dysfunction, as reported in earlier studies.^19^, ^20^ Subdomains were considered positive if score >0.^21^

The COMPASS‐31 was translated from English to Arabic by a bilingual translator and expert opinion was taken to assess its validity. A pilot testing of the translated questionnaire was performed on 30 participants to ensure that all questions were clear and could be understood by the participants. Items were evaluated for their internal consistency using Cronbach's α coefficient (*α* = 0.85).

Ethical approval was obtained from the Institutional Review Board (IRB) at the University of Assiut, Egypt (IRB Approval Number: 579/11/21). All patients gave their written informed consent before participation in the study.

### Statistical analysis

All statistical analyses were performed using IBM SPSS Statistics version 25 (SPSS Inc., Chicago, IL, USA). Categorical data were presented as numbers and percentages. Continuous data were tested for normality using the Kolmogorov–Smirnov test. Normally distributed continuous variables were summarized as mean and standard deviation (SD), while not normally distributed variables were presented as median and interquartile range (IQR). Differences in means were tested for statistical significance with independent‐samples Test; Mann–Whitney test was used as a nonparametric alternative test for not normally distributed variables. Spearman's correlation was used to test for association between COMPASS‐31 and other continuous study variables. *p* < 0.05 was considered statistically significant.

## Results

**Figure 1:**
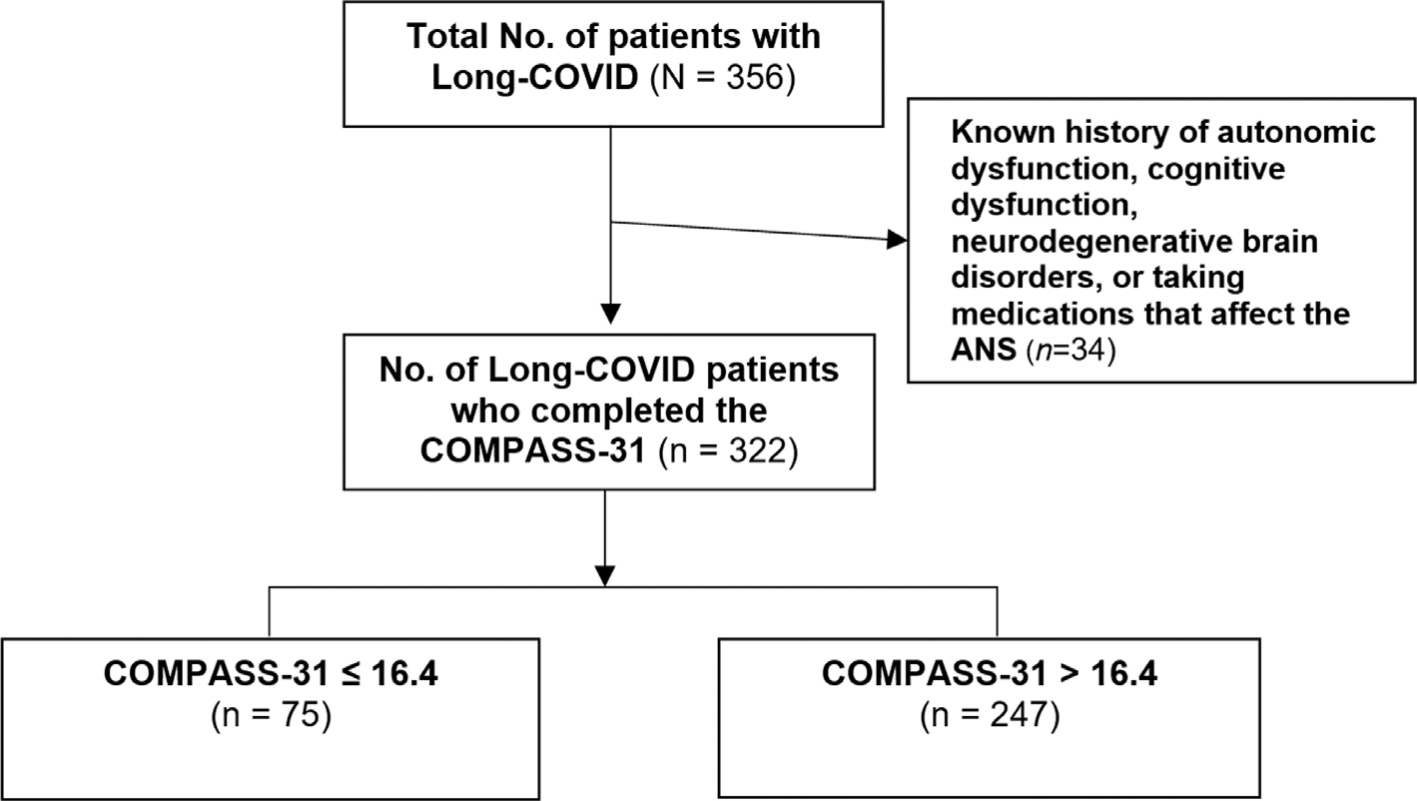
Flowchart of the study population.

We first included 356 patients in the data collection; after excluding those with a known history of autonomic dysfunction, cognitive dysfunction, neurodegenerative brain disorders, or those who are taking medications that affect the ANS, 322 participants were included in the final analysis (Fig. 1). Among these, 87 patients (27%) were male and 235 were female (73%). Age ranged from 18 to 74 years, with a mean of 35.9 years. Most patients (90.4%) had a diagnosis of SARS‐CoV‐2 for more than 12 weeks, with a mean duration of 41.9 weeks (Table 1).

**Table 1:** Basic characteristics of long COVID‐19 participants (*n* = 322).

Characteristics	*n* (%), or mean ± SD
Age (years)	
<40	211 (65.5%)
≥40	111 (34.5%)
Mean ± SD	35.92 ± 11.92
Min–max	18–74
Gender	
Male	87 (27%)
Female	235 (73%)
Long‐COVID duration	
4–12 weeks	31 (9.6%)
>12 weeks	291 (90.4%)
Mean ± SD	41.9 ± 18.3

The total weighted score of COMPASS‐31 ranged from 0 to 76.73 with a median of 26.3. The percentage of patients with a total weighted score of COMPASS‐31 >16.4, was 76.7%. The median and range of each COMPASS‐31 subdomain score are presented in Table 2. The percentage of patients with positive COMPASS‐31 subdomains indicated that the most involved domains were GI, secretomotor, OI, and pupillomotor with 91.6%, 76.4%, 73.6%, and 68.3%, respectively (Table 2).

**Table 2:** The distribution of COMPASS‐31 weighted scores and its correlation with age and long‐COVID duration (*n* = 322).

COMPASS‐31 [weighted scores range]	Median (range)	COMPASS‐31 score >16.4	Spearman's correlation	
			Age (years)	Long‐COVID (week)
COMPASS‐31 total score [0–100]	26.3 (0–76.7)	76.7%	0.011	0.263**
COMPASS‐31 subdomains scores	0	>0		
Orthostatic intolerance [0–40]	16.0 (0–36)	73.6%	0.159*	0.313**
Vasomotor [0–5]	0 (0–5)	19.9%	0.044	0.089
Secretomotor [0–15]	4.9 (0–13)	76.4%	−0.069	0.008
Gastrointestinal [0–25]	5.4 (0–21)	91.6%	−0.227**	−0.054
Bladder [0–10]	78.0 (0–7)	44.4%	−0.021	0.032
Pupillomotor [0–5]	1.3 (0–5)	68.3%	−0.096	0.012

** Statistically significant at *p* < 0.001.

* *p* < 0.01.

There was a significant positive but weak correlation between COMPASS‐31 total score and long‐COVID duration (*r* = 0.263, *p* < 0.001). No statistically significant correlations existed between the COMPASS‐31 total score and patients' age (*r* = 0.011, *p* = 0.842) (Table 2).

**Figure 2:**
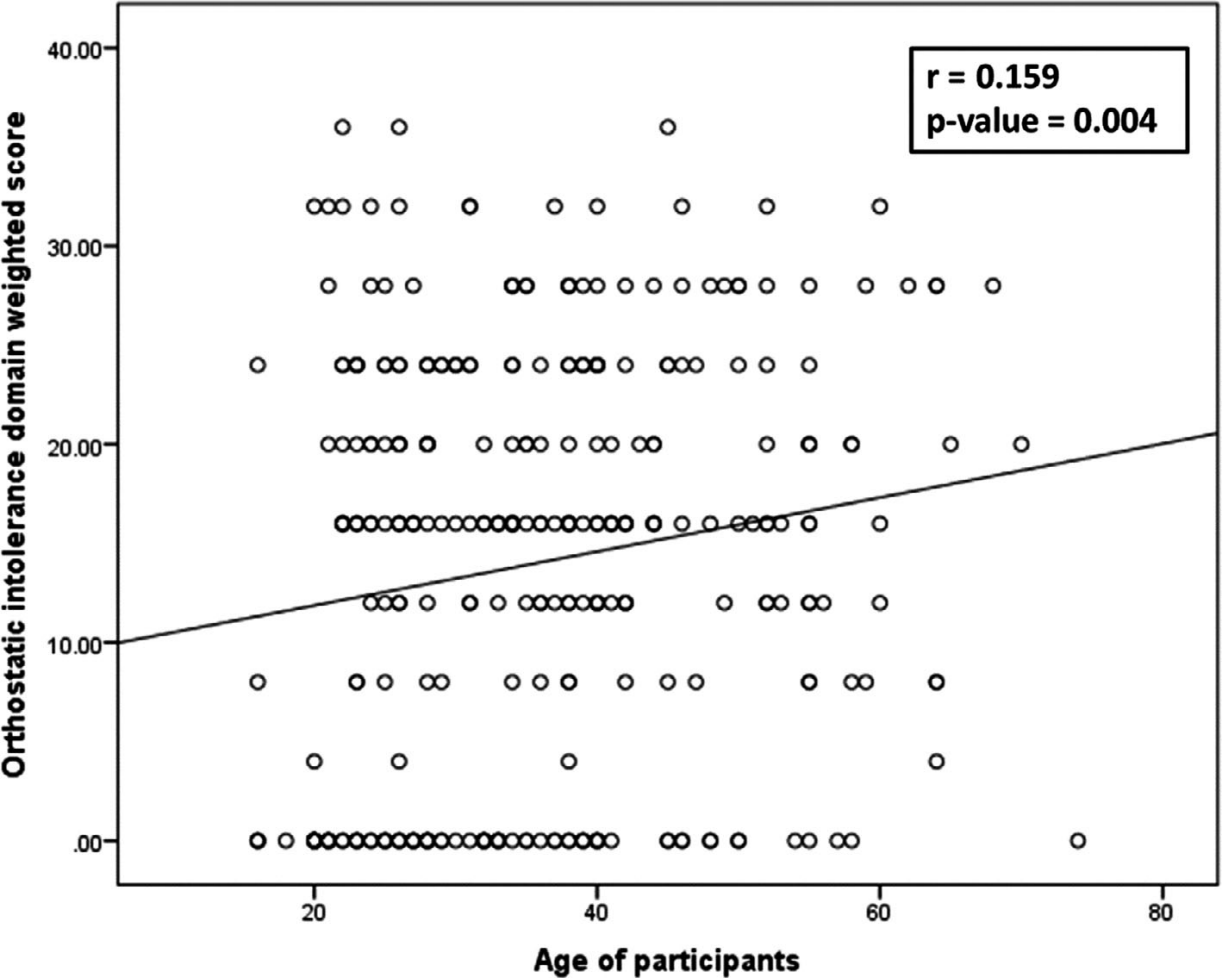
Correlation between age of participants and orthostatic intolerance domain weighted score of COMPASS.

Regarding COMPASS‐31 subdomains scores, there was a significant positive but weak correlation between OI domain score and long‐COVID duration (*r* = 0.313, *p* < 0.001), while other domains of COMPASS‐31 did not show a significant correlation. There was a significant negative but weak correlation between GI domain score and patients' age (*r* = − 0.227, *p* < 0.001). However, there was a positive correlation between OI domain score and patients' age (*r* = 0.159, *p* = 0.004). (Fig. 2) Other domains scores did not show any significant correlations with the patients' age (Table 2).

The COMPASS‐31 total score was not statistically significantly different between male and female or between patients aged above and below 40‐year‐old (*p* = 0.937, 0.515, respectively) (Table 3).

**Table 3:** The distribution of COMPASS‐31 total weighted scores and ANS dysfunction by patients' characteristics (*n* = 322).

Patients' characteristics	*N*	COMPASS‐31 Total score [0–100]		COMPASS‐31 score >16.4 *n* (row %)		
		Median (range)	*p*‐value	Yes (*n* = 247)	No (*n* = 75)	*p*‐value
Age (years)						
<40	211	27.8 (0–76.7)	0.515	157 (74.4%)	54 (25.6%)	0.178
≥40	111	24.5 (3.1–52.2)		90 (81.1%)	21 (18.9%)	
Mean ± SD				36.7 ± 11.8	33.5 ± 12.2	
Gender						
Male	87	28.0 (0–57.0)	0.937	67 (77.0%)	20 (23.0%)	0.938
Female	235	26.5 (0–76.7)		180 (76.6%)	55 (23.4%)	
Long‐COVID duration						
4–12 weeks	31	11.4 (0–40.0)	<0.001**	8 (25.8%)	23 (74.2%)	<0.001**
>12 weeks	291	28.6 (0–76.7)		239 (82.1%)	52 (17.9%)	
Mean ± SD				46.2 ± 14.8	26.8 ± 20.0	

** Statistically significant at *p* < 0.001.

Two hundred forty‐seven patients (76.7%) have a high total weighted score of COMPASS‐31 >16.4. When comparing the basic characteristics of participants between patients with COMPASS‐31 >16.4 and those with score <16.4, patients with a score >16.4 had a significantly longer duration of long‐COVID syndrome (46.2 vs. 26.8 weeks, *p* < 0.001). However, there was no statistically significant association between COMPASS‐31 score >16.4 and patients' age or gender (Table 3).

## Discussion

Our study highlights the high prevalence of symptoms of dysautonomia in long‐COVID syndrome patients. About 76.7% of our patients have high COMPASS‐31 score >16.4, which is used before as a cutoff to suggest initial autonomic nervous system dysfunction.^19^, ^20^ Our findings conform to many previous studies demonstrated autonomic dysfunction as one of the common features of long‐COVID syndrome.^7^, ^12^, ^13^, ^14^, ^15^, ^17^, ^22^, ^23^ High prevalence of autonomic dysfunction symptoms in long‐COVID (61.1%) was also seen in a recent study however COMPASS‐31 score >13.25 was used as a cutoff to suggest autonomic nervous system dysfunction.^17^

Not much literature that discusses the etiology and rational of autonomic dysfunction associated with COVID‐19, but few mechanisms have been suggested. SARS‐CoV infection in mice downregulates the ACE2 expression.^24^ Change in ACE2 expression or function can lead to blood pressure changes as ACE2 catalyzes the hydrolysis of vasoconstrictors (angiotensin I or II) into vasodilators (angiotensin 1–9 and 1–7, respectively) resulting in lowering the blood pressure.^25^ Downregulation of the ACE2 expression is also coupled with tumor necrosis factor α production and affects neuronal regulation of inflammation. Inflammatory cytokines associated with SARS‐CoV infection activate the afferent sensory vagus nerve, which leads to activation of the efferent vagus nerve to control the inflammation by inhibiting the production of inflammatory cytokines in the macrophage.^26^ Hyperactivation of the vagus nerve also downregulates the expression or activity of ACE2.^27^, ^28^ In essence, The interaction of SARS‐CoV‐2 with ACE2 can result in dysregulation of the potent renin–angiotensin system that leads to impairment of cardiovascular regulation and parasympathetic tone.^29^ Positron emission tomography findings have suggested possible COVID‐19 neurotropism through the olfactory bulb with impairment of other areas of the brain that control the autonomic nervous system, including the thalamus, hypothalamus, and brain stem^30^, ^31^ Afferent baroreflex failure with impairment of heart rate variability also factor in the autonomic dysfunction.^32^, ^33^ COVID‐19 led to a cytokine storm with the release of large number of pro‐inflammatory cytokines that can affect various body organs.^34^ Because of COVID‐19 and hyperinflammatory release, sympathetic hyperactivation occurs and contributes to the cardiovascular and gastrointestinal systems dysfunction.^35^ For example, sympathetic hyperactivation can cause tachyarrhythmias, hypertension, hyperhidrosis, reduced intestinal peristalsis, and lead to serious complications including myocardial injury. Moreover, sympathetic activation induces pro‐inflammatory cytokines that contribute to the hyperinflammatory response.^29^

COMPASS‐31 score was higher in patients with a diagnosis of SARS‐CoV‐2 for more than 12 weeks (*p* = <0.001) and patients with a high COMPASS‐31 score (>16.4) had longer post‐COVID duration than those with a low score (46.2 vs. 26.8 weeks). We postulate that in addition to the possible mechanisms of autonomic dysfunction associated with COVID‐19 explained above, they may be an additional component of deconditioning and increased anxiety with longer duration of post‐COVID‐19 illness that worsens the symptoms. The role of physical and psychosocial rehabilitation as part of the management of post‐COVID autonomic dysfunction needs further investigation.

In this study, we did not find significant differences in COMPASS‐31 score between male or female or between patients above 40‐year‐old compared to those below 40 (*p* = 0.937, 0.515, respectively). The same findings hold true in patients with high COMPASS‐31 score (>16.4) compared to those with low score (26.8 ± 20.0 weeks).

The most affected COMPASS‐31 domains with a score above 0 are the GI, secretomotor, and OI. There was a negative but weak correlation between GI domain score and age of participants (*r* = −0.227, *p* < 0.001). ACE2 is highly expressed in the esophagus and enterocytes in the colon and ileum.^36^, ^37^ Therefore, COVID‐19 could infect and replicate in ACE2 mature enterocytes.^38^ Thus, GI involvement is common in COVID patients and lead to multiple symptoms including vomiting, nausea, diarrhea, abdominal pain, constipation, loss of appetite, and anorexia.^39^, ^40^, ^41^ These symptoms manifest early with COVID‐19 or as part of long‐COVID syndrome.^36^ Diarrhea and anorexia were found to be the most significant GI symptoms after COVID‐19 in a meta‐analysis conducted with 31 studies that included 4682 patients.^36^

There was a positive but weak correlation between OI and post‐COVID duration (*r* = 0.313, *p* < 0.001); and a positive correlation between OI and age of participants (*r* = 0.159, *p* = 0.004. OI reported to be the most common autonomic dysfunction findings in patients referred to mayo clinic autonomic laboratory for autonomic reflex screen testing and in fact about 93% of those patients' reported lightheadedness.^22^ Increasing age can be associated with many factors that impair the normal blood pressure regulation upon standing, including decreased baroreflex sensitivity, decreased alpha 1‐adrenergic vasoconstrictor response to sympathetic stimuli, decreased renal salt and water conservation, increased vascular stiffness, and reduced left ventricular diastolic filling^42^, ^43^, ^44^, ^45^; impairment of orthostatic blood pressure regulation with increasing age due to the aforementioned factors will likely explain the positive correlation between OI domain and age of the participant.

The involvement of secretomotor domain was common in our study and agrees with previous reports.^22^, ^46^ In mayo clinic review of autonomic dysfunction following COVID‐19, sudomotor function evaluated by QSART and thermoregulatory sweat test was abnormal in 36%.^22^

### Study limitations

This study has limitations. First, the study is observational and therefore we can only report an association between the long‐COVID syndrome and symptoms of dysautonomia and we cannot report a solid conclusion regarding the causative relationship or the underlying mechanism. Second, we based our evaluation on the questionnaire that is affected by subjective interpretation and emotional components, which may create a response bias in addition to a recall bias. However, we used COMPASS‐31 questionnaire, which is a validated and well study tool for evaluating autonomic symptoms. Third, the objective evaluation of autonomic dysfunction using autonomic testing such as autonomic reflex screen including head‐up tilt, Valsalva, heart rate response to deep breathing, and quantitative sudomotor axonal reflex was not performed to objectively confirm the autonomic dysfunction.

## Conclusion

Symptoms of dysautonomia are common in long‐COVID syndrome patients. The most affected COMPASS‐31 domains are GI, secretomotor, and OI. There is a positive correlation between OI domain score and patients' age and there is a positive but weak correlation between COMPASS‐31 score and post‐COVID duration. Future studies will be needed for further evaluation of the proper management of autonomic dysfunction in patients with long‐COVID syndrome.

### Statements and Declarations

• On behalf of all authors, the corresponding author states that there is no conflict of interest.
• All human studies have been approved by the appropriate ethics committee and have therefore been performed in accordance with the ethical standards laid down in the 1964 Declaration of Helsinki and its later amendments; and the specific national laws have been observed.
